# Occupational and Individual Determinants of Work-life Balance among Office Workers with Flexible Work Arrangements

**DOI:** 10.3390/ijerph17041418

**Published:** 2020-02-22

**Authors:** Sofie Bjärntoft, David M. Hallman, Svend Erik Mathiassen, Johan Larsson, Helena Jahncke

**Affiliations:** Department of Occupational Health Sciences and Psychology, University of Gävle, 801 76 Gävle, Sweden; david.hallman@hig.se (D.M.H.); svenderik.mathiassen@hig.se (S.E.M.); johan.larsson@hig.se (J.L.); helena.jahncke@hig.se (H.J.)

**Keywords:** work-life balance, autonomy, job resources, job demands, work control

## Abstract

Flexible work arrangements permitting workers to work anytime and anywhere are increasingly common. This flexibility can introduce both challenges and opportunities for the organisation, as well as for worker work-life balance (WLB). This cross-sectional study aimed to examine the extent to which occupational factors (organizational, leadership and psychosocial) and individual work-related behaviours (over-commitment, overtime work and boundary management) are associated with WLB, and whether these associations are modified by the perceived level of flexibility at work (i.e., control over when, where, and how to do the work). In total, 2960 full-time office workers with flexible work arrangements at the Swedish Transport Administration participated. Associations were determined using linear regression analyses with adjustment for covariates. The strongest negative associations with WLB were found for over-commitment, quantitative job demands, expectations of availability, and overtime work. Strongest positive associations were found for boundary management, information about organizing work, social support, and relation-oriented leadership. Perceived flexibility was positively associated with WLB, and interacted with several of the examined factors, buffering their negative associations with WLB. Results suggest that WLB can be promoted by organizational initiatives focusing on minimizing excessive job demands, increasing psychosocial resources, supporting boundary management, and enhancing perceived flexibility.

## 1. Introduction

Developing sustainable and health-promoting work is an uncontested challenge in occupational health sciences [1,2,3]. Work-life balance (WLB) is a key factor influencing worker health and well-being, as demonstrated by its inclusion as an important element in the conceptual ideas of ‘attractive work’ and ‘the good work’ [4,5]. WLB is a multi-dimensional concept that has been defined in different ways in previous studies [6,7]. The definition used in the present study is based on that suggested by Kalliath and Broughs [6] (p. 326), that is; “work-life balance is the individual perception that work and non-work activities are compatible and promote growth in accordance with an individual´s current life priorities”. How to attain a good WLB has gained more attention over the last two decades [8]. One possible explanation is the dramatic increase in digitization, including an increased use of information and communication technologies (ICT). This has enabled organisations to offer flexible work arrangements that may create both opportunities and challenges for workers WLB [9,10,11]. Depending on the organisation, the work tasks, and the needs of the workers, flexible work arrangements can permit: flexibility in time, referring to autonomy with respect to working hours; flexibility in space, referring to opportunities to select more than one working location (e.g., work from home); and flexibility in performance, referring to how the work can be performed [12].

Flexible work arrangements have become wide-spread, and according to The Organisation for Economic Co-operation and Development [13], in 2016, three-quarters of workers in Europe had some type of work-time flexibility, with Swedish workers having the next highest rate of working from home. Flexible work arrangements can be beneficial for WLB by increased autonomy to combine work and personal life [13,14]. A good WLB can lead to improved worker well-being [13], positive job-related attitudes, job satisfaction, organizational commitment [15], job performance, and career development [7]. Flexible work arrangements may, however, also present considerable challenges for the individual in setting boundaries between work and personal life; as limited success in doing so may result in conflicts between work and personal life [16,17]. Ultimately, a poor WLB may lead to adverse health-related outcomes, such as: self-reported sickness absence [18], job- and life dissatisfaction, job burnout, depression, irritability, fatigue, and increased blood pressure and cholesterol levels [7]. These results suggest that flexible work arrangements can have both positive and negative effects on WLB and illustrates the need for research identifying occupational factors and individual behaviours that have a substantial impact on how WLB is experienced by workers with flexible work arrangement.

Worker success in achieving a good WLB in flexible work arrangements is likely affected (either positively or negatively) by factors related to work [19,20]. At an organizational level, clear guidelines and information about how to work flexible is particularly important for an effective use of flexibility [21]. However, this has not been investigated in relation to WLB, but insufficient organizational conditions for flexible work arrangements may deter a satisfying combination of work and personal life. Previous research [22,23] also suggests that the physical work facility, such as office type, may influence worker WLB. For instance, activity-based offices may promote the balance between work and personal life by increasing autonomy in where and how the work can be performed [22]. However, there is limited research on the association between office type and WLB in populations with flexible work arrangements.

Leadership behaviour has also been suggested to be an important determinant of worker WLB [24,25]. We examined leadership using the three-dimensional leadership behaviour model, first suggested by Ekvall and Arvonen [26] and later developed by Yukl [27,28,29]. This well-established model, distinguishesing between leadership behaviours that are relation-oriented (i.e., focusing consideration, trust, and socialization), structure-oriented (i.e., focusing clear goals, instructions, and follow-up of performed work) and change-oriented (i.e., focusing new work methods, visions, and development), has previously been used to examine the effect of leadership on both individual and organizational outcomes. All three leadership behaviours may increase efficiency, job satisfaction and health among workers [30,31,32]. Recently, the model has been used in the context of WLB, and relation-oriented behaviour was found to be a key for a good WLB, regardless of the extent of job and family demands [24]. However, lack of research investigates the effect of all three leadership behaviours on workers WLB, particularly the structure- and change-oriented dimensions, and evaluating the behaviour effects in the context of flexible work arrangements. Flexibility at work may prevent managers from practicing certain leadership behaviours (e.g., being supportive and visible) that would otherwise promote worker WLB. 

Poor psychosocial working conditions including high job demands, time pressures and expectations of availability have been shown to adversely affect WLB [33]. Excessive job demands can lead to work outside of regular working hours, which may reduce time and energy for private activities outside of work, subsequently leading to poor WLB [20]. In contrast, factors related to the psychosocial work environment can also have a positive effect on WLB. For example, a supportive culture that encourages flexibility without feeling guilt for being ‘off’ during traditional working hours may increase WLB [19]. In addition, managerial support may mitigate feelings of guilt when employees are on sick leave, which in turn may reduce presenteeism [34]. Thus, a good social community at work and social support from colleagues appears important in maintaining a good WLB in flexible work arrangements [19]. 

In addition to occupational factors, WLB is likely influenced by individual behaviours. An individual behaviour that may challenge WLB is performing excessive overtime work, which can include frequently bringing work home, answering emails outside of regular working hours, and working during weekends and holidays [33]. This behaviour may be common among workers with flexible work arrangements, and it results in longer working hours and in turn poorer WLB [33]. Another example of an individual behaviour that may adversely affect WLB is over-commitment to work, defined as “a set of attitudes, behaviours and emotions that reflect excessive striving in combination with a strong desire to be approved of and esteemed” [35] (p. 55). Previous research [20] has found over-commitment to be strongly associated with poor WLB. 

A key factor in promoting worker WLB may be perceived flexibility of work, i.e., the level of perceived control over when, where and how to do the work [12]. Having flexible work arrangements only showed a marginal positive effect on worker WLB, while perceived flexibility was strongly positively associated with WLB, likely due to the perceived opportunity to control work [14]. However, previous research has mainly focused on the interaction of flextime use on the association between work-family conflict and job satisfaction and organizational commitment [36]. No known studies have investigated the extent to which perceived flexibility interacts with a broad range of occupational factors and individual behaviours to determine WLB among workers in organisations with flexible work arrangements. 

In summary, both occupational factors and individual behaviours related to work contribute to shaping a worker’s WLB. The trade-off between negative and positive determinants of WLB is paramount for supporting organisations with flexible work arrangements in developing interventions that can effectively prevent poor WLB and promote a good WLB, but it has not been addressed in previous research. 

### 1.1. Theoretical Background

The present study is grounded in a theoretical framework including Job Demands-Resources (JD-R) model and Boundary Theory, which may explain the potential association between negative and positive determinants of WLB and the interaction of perceived flexibility. The JD-R model is a well-established theoretical model, in which occupational factors are classified either as job demands (e.g., work pressure) or as job resources (e.g., social support) [37,38,39]. These two domains create two independent processes; one that is energy-driven, where excessive job demands may lead to negative health outcomes, and one that is motivation-driven, where good resources may lead to organizational commitment and dedication. These two processes can also interact in predicting well-being, for example by resources moderating a negative association between job demands and strain, or by job demands leading to a stronger positive association between resources and motivation [39]. Recently, this model has been used in the context of WLB [24,40]. High job demands and limited job resources tend to increase strain on workers, which can lead to reduced WLB, whereas a good WLB can be achieved in spite of high job demands if the individual receives sufficient job resources [41]. Therefore, we propose that a high level of perceived flexibility can be a resource in the context of worker WLB, acting by buffering negative effects of occupational factors and individual behaviours on WLB. 

The effects of occupational factors and individual behaviours on WLB can be explained by the worker’s ability to set boundaries between work and personal life, framed in Boundary Theory. According to this theory, individuals seek to create and maintain physical, cognitive and behavioral boundaries between work and personal life, in order to simplify everyday life [42]. The extent to which an individual manages to achieve boundaries depends on the individual’s boundary management and preferences regarding whether work and personal life are separated (“segmentation”) or intertwined (“integration”) [33]. Workers may differ in boundary management and it is reasonable to expect that they will therefore experience different degrees of WLB even under the same working conditions [33]. A modifying role of perceived flexibility is also consistent with Boundary Theory [42]. Increased autonomy over work may make it easier to achieve preferred boundary settings, which, in turn, can alleviate a negative influence on WLB of a poor working environment. More autonomy at work may increase opportunities to handle stressful situations, including a poor psychosocial work environment [34]. However, this proposed buffering role of perceived flexibility on associations between occupational factors, individual behaviours and WLB in flexible work arrangements has not been addressed in previous research.

### 1.2. Aim and Research Questions

The aim of the present study was to examine the extent to which selected occupational factors and individual behaviours are associated with work-life balance (WLB) among office workers with flexible work arrangements, and whether such associations are modified by perceived flexibility at work. 

**Figure 1:**
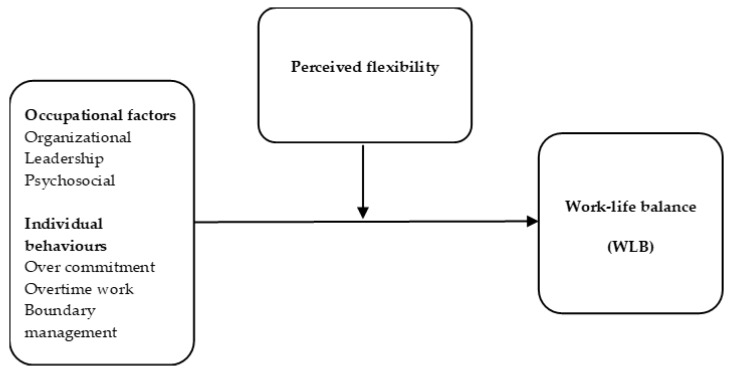
Model associating occupational factors and individual behaviours with work-life balance (WLB) (research question 1), with perceived flexibility as a modifier (research question 2).

We formulated two research questions (Figure 1):

(1) To what extent are selected occupational factors (in the organizational, leadership and psychosocial domains) and individual behaviours associated with WLB among office workers with flexible work arrangements?
(2) To what extent are any such associations modified by the level of perceived flexibility at work?

## 2. Materials and Methods

### 2.1. Study Setting and Sample

This cross-sectional questionnaire study was conducted at the Swedish Transport Administration, a large governmental agency in Sweden, as part of a larger research project. The Human Resources department of the agency identified all workers with flexible work arrangements and handed out their job email addresses to the research group. In October 2016, all workers and managers with flexible work arrangements received an email from the research group containing information about the study, an invitation to participate, and a link to a comprehensive web-based questionnaire. Workers had two months to complete the survey, and email reminders were sent at two-week intervals. Respondents were included in the study if they had a work contract allowing for flexible work arrangements (i.e., flextime or non-regulated working hours). Exclusion criteria were part-time work (since working hours per week may affect worker WLB), sick leave and parental leave. Of the 4900 eligible workers receiving the questionnaire, 3259 responded (response rate 66.5%). Of these, 284 respondents were excluded due to the exclusion criteria, and 15 respondents were excluded because they had not answered the WLB question (see Section 2.2). 

The final sample consisted of 2960 participants and comprised 2312 front line workers (78.3%) and 641 (21.7%) managers and supervisors (e.g., project leaders). All workers who were asked to participate in the study had some type of flexible work arrangements, whereby 70.4% reported to have non-regulated working hours and 28.5% had flextime (1.1% had other arrangements). Of the whole sample (*n* = 2960), 56% were men, 38% were woman, 3% of the participants did not want to categorize themselves as either man or women and 3% were missing. The average age was 47.9 years (SD 9.7; range 17–68). The highest education level was college/university for a majority of the participants (62.8%), 27.9% had high school, and 1.4% primary school. On average, the participants had been employed in the organisation for 14.7 years (SD 11.0; range 2–51), and in their current position for 6.3 years (SD 5.4; range 2–50). All participants signed an informed consent prior to responding to the questionnaire. The study was approved by the Regional Ethical Review Board in Uppsala (Dnr 2016/085).

### 2.2. Study Measures

The present study focused questions addressing occupational factors and individual behaviours pertaining to flexible work, WLB, organizational factors, leadership behaviours, psychosocial conditions, and work-related individual behaviours. The questionnaire contained both modules from previously validated questionnaires (Copenhagen Psychosocial Questionnaire (COPSOQ) [43] and Siegrist [44], and questions developed specifically for this study to assess additional aspects relevant to flexible work and WLB. Prior to the study, the entire questionnaire was validated using “think aloud interviews” and principal component analysis (PCA) [45]. In the present study, we used factor analysis to construct indices of particularly relevant questions in the context of our research questions. We used Varimax rotation with the following limits; Kaiser-Meyer-Olkin (KMO) = >0.6, Bartlett’s test *p* ≤ 0.05, percent of variance >60, Eigen-value >1 and rotated component value >0.3. All indices in the study had Cronbach Alpha values between 0.71 and 0.90. 

An overview of the measures of all independent variables used in this study is provided in Table 1. The dependent variable work-life balance (WLB) was measured using a single question addressing the level of satisfaction with WLB, modified from Hansson [46]: “how satisfied are you with your work-life balance?” rated on a five-point scale from 0 (not at all) to 4 (to a very high extent). A detailed description of all questions included in each variable is available in the Supplementary Materials.

We selected covariates based on a likely association with the investigated occupational factors and individual behaviours, and with WLB [47,48,49]. Covariates included age (year of birth); level of education (primary school, high school, vocational school and college/university); gender (woman, man, do not want to categorize); years of employment within the organisation (years); position in the organisation (manager or employee); and children at home (yes or no). 

### 2.3. Statistical Analyses

All analyses were performed using SPSS version 24 (IBM, Armonk, NY, USA). Means and standard deviations (SD) (continuous variables) or frequencies and percent (categorical variables) were used for descriptive purposes. We used multiple linear regression analysis to investigate associations between occupational factors and individual behaviours (independent variables), and WLB (dependent variable), with adjustment for covariates. Hierarchical regression models were constructed, first by adjusting for all covariates (model 1), then adding occupational factors and individual behaviours, one after the other, modelled as independent variables (model 2). Thus, the main effect of each occupational factor and individual behaviour was determined using model 2. We then investigated the possible effect modification by perceived flexibility for each occupational factor and individual behaviour, one after the other, in a new set of models (model 3). In each case, model 3 included the main effects of perceived flexibility and the factor of interest, as well as the two-way interaction between perceived flexibility and the factor of interest, calculated by multiplying the factor with perceived flexibility. Statistically significant interactions were further inspected by plotting the linear relationship between the occupational factor/individual behaviour and WLB for tertiles of perceived flexibility. For each model, we determined an effect estimate for the independent variable (B), the standard error (SE) of B, the p-value, and the change in explained variance (i.e., ∆R^2^) from the preceding model. Effect sizes were classified as small (R^2^ ≤ 0.02), medium (0.02 < R^2^ < 0.26), and large (R^2^ ≥ 0.26), modified after Cohen [53]. 

In all regression analyses, we assessed linearity and normal distribution of the residuals, and found no critical violations of these assumptions. We determined the extent of multicollinearity among the independent variables using collinearity diagnostics with VIF limit set at >5 [54]; no variables were excluded due to violations. Common method bias was checked by Harman’s test (cut point 30%), and we found no critical bias that would affect the eventual results (19.5% variance attributable to common-methods effects).

Since WLB may be experienced differently by men and women, and gender may modify associations between working conditions and WLB [55,56], we performed a sensitivity analysis by re-running the regression models above including two-way (factor x gender) and three-way (factor x flexible work x gender) interactions. We examined statistically significant interactions (*p* < 0.05) further, using gender-stratified analyses. 

## 3. Results

### 3.1. Descriptive Statistics

On average, participants reported a WLB of 2.93 (SD = 1.00) and a perceived flexibility at work of 2.67 (SD = 0.90). Descriptive statistics for all occupational factors and individual behaviours are shown in Table 2. 

### 3.2. To What Extent are Occupational Factors and Individual Behaviours Associated with Work-life Balance?

The covariates included in model 1 explained 2% (∆R^2^ = 0.020) of the variance in WLB. Statistically significant associations with WLB were found for gender (ref female, B = 0.12, *P* = 0.004), university degree (ref other, B = 0.09, *P* < 0.001), children at home (ref, no children, B = −0.14, *P* < 0.001), managing position (ref employee, B = 0.12, *P* = 0 .008), and years of employment (B = −0.004, *P* = 0.048). 

For model 2, statistically significant associations with WLB were found for all occupational factors and individual behaviours except flexible work arrangements and office type (Table 3). Factors with the strongest negative association with WLB were over-commitment (∆R^2^ = 0.317) and quantitative job demands (∆R^2^ = 0.227), with large and medium effect sizes, respectively. Pronounced negative associations were also found for expectations of availability (∆R^2^ = 0.105), overtime work (∆R^2^ = 0.091) and expectations to work more than agreed (∆R^2^ = 0.088), however with small effect sizes. The factor showing the strongest positive association with WLB was boundary management (∆R^2^ = 0.316), which also had a large effect size. Pronounced positive associations, if with smaller effect sizes, were also found for information about organizing work (∆R^2^ = 0.089), social support from colleagues (∆R^2^ = 0.080), relation-oriented leadership (∆R^2^ = 0.071), social community at work (∆R^2^ = 0.070), and influence at work (∆R^2^ = 0.059). Perceived flexibility (∆R^2^ = 0.065) showed a small positive association with WLB (small effect size). Associations for all factors after adjustment for covariates are reported in Table 3 (model 2, main effects columns). 

### 3.3. To What Extent are Associations of Occupational Factors and Individual Behaviours with Work-life Balance Modified by the Level of Perceived Flexibility?

Model 3 showed that perceived flexibility significantly modified the effects of factors and behaviours from all categories (Table 3, model 3). The positive interaction effects indicate that the negative associations of expectations of availability, expectations to work more than agreed, and overtime work with WLB, were less pronounced among workers with high levels of perceived flexibility. Higher levels of perceived flexibility even led to less pronounced positive associations between the factors information about organizing work, structure- and change-oriented leadership, and social support from colleagues, with WLB. The interactions above all attenuated the main effect of the factor of concern. The only factor for which perceived flexibility amplified the association with WLB was flextime (referencing non-regulated working hours). In general, interaction effects with perceived flexibility were small, resulting in only marginal increases in explained variance (∆R^2^ at the most 0.008; Table 3).

### 3.4. Moderation by Gender

The analyses addressing the extent to which gender influenced associations did not reveal any statistically significant gender interactions, except for expectations of availability (interaction B = −0.08, SE = 0.02, *P* ≤ 0.001, ∆R^2^ = 0.003) and overtime work (B = −0.02, SE = 0.01, *P* ≤ 0.001, ∆R^2^ = 0.007). Stratified analyses showed that the effects of both factors were more pronounced among women than among men.

## 4. Discussion

This study identified occupational factors and individual work-related behaviours as potential determinants of WLB among office workers with flexible work arrangements. Specifically, we found that over-commitment and quantitative job demands were strongly associated with reduced WLB (large and medium effect size), while boundary management was strongly associated with better WLB (large effect size). Perceived flexibility was positively associated with WLB and interacted with several occupational factors and individual behaviours, suggesting a buffering role of perceived flexibility. 

Over-commitment and high job demands showed strong negative associations with WLB. For each unit of increase in over-commitment (scale 1–4) and demands (scale 0–4), WLB was reduced by 0.9 and 0.7 units, respectively. This is in line with a previous cross-sectional study [20] that also found that these factors were strongly associated with increased work-life conflict, which suggests that over-commitment and high job demands may be a threat against WLB in populations with flexible work arrangements. One explanation may be that high commitment to work and job demands can both reduce workers’ time and energy to engage in family and personal life after the workday, which may decrease their satisfaction with WLB [20].

Expectations of availability and performing overtime work were also negatively associated with WLB, although with small effect sizes. One explanation may be that expectations of availability outside regular working hours can make it difficult to disconnect from work after the workday, which in turn can result in longer working hours and reduced WLB. Overtime work was reported by many of the office workers in the present study (mean time of 2 h/week), which agrees with previous research [33] reporting overtime work to be a common behaviour in workers with flexible work arrangements. 

Boundary management showed a strong positive association with WLB. An increase in perceived boundary management of one unit predicted an increased WLB of 0.6 units (both scales 0–4). A high degree of boundary management can result in improved management of work and family demands, which in turn can lead to a good WLB [33,42]. Our results agree with a previous study by Mellner et al [32] reporting boundary management to be positively associated with WLB [33]. However, the study found that less boundary management reduced WLB specifically among segmenters (i.e., individuals preferring to separate work and personal life), but not among integrators (individuals preferring to mix work and personal life), even though the latter group worked more overtime [33]. 

Other factors that were significantly associated with a good WLB included information about organizing work, leadership behaviours, social community at work, social support from colleagues, a culture encouraging flexible work, influence at work, and clear expectations of availability. These factors at an organizational level thus appear important to consider for managers wishing to promote WLB in flexible work arrangements. Leadership behaviours, in particular relation-oriented leadership, may promote a supportive work environment and make it easier to cope with excessive job demands and set proper boundaries between work and personal life [24]. 

Our finding of a positive association of perceived flexibility with WLB corroborates results in several previous studies [11,14], but contradicts others, suggesting that schedule flexibility increases work-family conflicts [16], and that work from home increases work pressure and work-life conflicts [17]. We found that perceived flexibility buffered the negative effects of several occupational factors and individual behaviours on WLB, including expectations of availability, work more than agreed, and overtime work. Thus, the negative associations were attenuated among workers who experienced a high degree of perceived flexibility. However, we also found that perceived flexibility attenuated the effects of factors with positive associations with WLB, including, structure- and change-oriented leadership behaviour, social support, and information about organizing work, among workers who had a high level of perceived flexibility. These findings may indicate that factors promoting WLB can be less important for workers who also experience a high degree of flexibility at work. For example, we found an interaction effect on the positive association between structure- and change-oriented leadership behaviours and WLB, which indicates that these types of leadership behaviours (but not relation-oriented leadership) may be particularly important to WLB when workers perceive less control over work. One explanation may be that relation-oriented leadership behaviour is important for employees’ WLB regardless of the level of flexibility. This is in line with previous research [24] showing that relation-oriented leadership behaviour is a key factor for employees’ WLB, regardless of the extent of job demands. Our findings indicate that the need for structure- and change-orientation is reduced in employees with high self-control, suggesting that they also take ownership of their work situation, also referred to as employeeship [57]. Thus, employees with flexible work arrangements experiencing low flexibility may be in particular need of a leadership providing clear goals and instructions (structure-oriented behaviour) or new work methods and development (change-oriented behaviour) to obtain a good WLB.

In contrast, over-commitment, high job demands, and boundary management did not show significant interactions with perceived flexibility. Thus, our results suggest that these factors affect WLB, and do so to a high extent (cf. Table 3), regardless of the level of perceived flexibility. 

Our study is, to our knowledge, the first to show a moderating effect of perceived flexibility on the associations between occupational factors and individual behaviours and WLB among office workers with flexible work arrangements. In contrast, previous research [36] reported that current flextime use amplified the negative association between work-family conflict and job satisfaction and organizational commitment. One possible explanation may be that perceived flexibility is more beneficial for worker WLB than flextime use, because it includes their perception of control over when, where and how to work, and not only whether flexibility is used or not [14].

Drawing on the JD-R model [37,38], our study identified several job demands (e.g., quantitative job demands, and expectations of availability) and job resources (e.g., information about organizing work, leadership behaviours, and social support from colleagues) which were associated with WLB. The JD-R model may also explain the observed interaction effects with perceived flexibility. A possible moderator according to the JD-R model is job autonomy (i.e., control over when and how to respond to job demands), which may act as a buffer on the association between high job demands and strain, where increased autonomy permits workers to better control stressful situations [37]. It therefore follows that a reasonable explanation for our findings is that flexibility at work acts in a similar way in reducing the negative effects of poor working conditions on WLB. A buffering effect of perceived flexibility is also consistent with the Boundary theory [42], which predicts that increased autonomy (i.e., control over work) will make it easier for workers to handle stressful and demanding situations and attain preferred boundary settings; this, in turn, reduces the negative effects of a poor psychosocial working environment. 

Previous research indicates that women often perceive lower WLB than men [56], and our results confirm that for office workers with flexible work arrangements. However, we found that gender rarely had an effect on associations between occupational or individual factors and WLB. The only exceptions were expectations of availability and overtime work, for which associations with WLB were more negative among women. This suggests that high expectations of being available and working overtime may impede WLB to a larger extent for women than for men. Gendered differences in WLB may, however, be more pronounced in other occupational populations. Thus, a study of work-life conflict in several European countries [58] found that women tended to use flexibility to combine work and personal life for a better WLB, while men rather increased their work commitment, resulting in larger work-family conflicts.

### 4.1. Strengths and Limitations

The present study addressed a comprehensive selection of factors of relevance to flexible work arrangements, including organizational, leadership and psychosocial domains, as well as individual behaviours. We considered these relationships in a large population (n = 2960) that had considerable contrast in perceived flexibility, which allowed us to assess even small effect sizes and interaction effects with good statistical certainty. In addition, we obtained a reasonably high response rate (66.5%) to our questionnaire, which strengthens the generalizability of the study findings within the organisation and reduces the risk of selection bias. However, the study also suffered some methodological limitations. The sample consisted of workers from one governmental agency in Sweden, which may limit generalizability to other organisations and other countries. Further, the use of self-reported information on occupational factors, individual behaviours, and WLB may have introduced common-methods variance. However, Harman’s test indicated a reasonably low risk of bias attributable to common-methods effects. Another limitation of the study may be that WLB was assessed using only one single item addressing satisfaction with WLB. Multi-item indices might have provided a more comprehensive description of WLB [59]. 

We used a cross-sectional study design, which essentially precludes inferences about causal associations. A cross-sectional study also precludes examination of long-term effects of changes in working conditions, which we emphasize as an issue for further research. We included each occupational factor and individual behaviour in separate regression models, and did not examine possible joint effects of several factors, with the exception of the factor, perceived flexibility, which we handled as an effect modifier. We suggest that the combinatory effects of occupational factors and individual behaviours on WLB as an issue for further research. Finally, the present population of office workers reported having a favorable working environment with high levels of both flexibility at work and of WLB, and, on average, good working conditions (cf. Table 2). It is therefore possible that other results can be seen in populations with less favorable working conditions.

### 4.2. Theoretical and Practical Implications

In keeping with the JD-R model [37,38], we hypothesized that perceived flexibility would modify associations of occupational factors and individual behaviours with WLB (Figure 1). We found empirical support confirming that a high level of perceived flexibility (control over when, where and how to work) led to less pronounced associations between these factors and WLB. Thus, our conceptual model (Figure 1) may be useful in further research examining effects of perceived flexibility at work. Second, our results add new knowledge on determinants of WLB that can inspire organisations in developing recommendations, interventions, and effective policies.

Our findings emphasize the importance of involving several levels within an organisation when developing initiatives focusing WLB. For instance, organizational interventions, such as, developing and clarifying policies and guidelines for flexible work, could be combined with interventions addressing leadership behaviours, and interventions in the psychosocial working environment, including clear expectations about availability and creating a culture encouraging flexible work. In addition, it may be important for organisations to focus on interventions at an individual level to reduce (self-selected) overtime work and over-commitment for example by increase competence in boundary management or offer education in effective methods to control work demands. We emphasize that many organisations currently offer flexible work arrangements, and this number will likely increase due to the increasing digitization and its influence on working life. Thus, even moderate initiatives leading to small effects may be important for WLB in the ever-growing population of workers with flexible work arrangements.

## 5. Conclusions

We found that work-related organizational, leadership, and psychosocial factors, and individual behaviours, were associated with work-life balance (WLB) among office workers with flexible work arrangements. Over-commitment to work (individual behaviour) and high job demands (psychosocial factor) were strongly negatively associated with WLB, while boundary management (individual behaviour) showed a strong positive association with WLB. Perceived flexibility (i.e., control over when, where and how to do the work) was positively associated with WLB, and appeared to buffer the impact of factors having a negative association with WLB. Our results suggest that organisations offering flexible work arrangements can promote WLB among workers by focusing on providing information regarding how to organize work, clear guidelines and policies for flexible work, clear expectations regarding availability outside of working hours, and a relation-oriented style of leadership. Overall, a high level of perceived flexibility, in particular in combination with other positive factors in the work environment, may support a good WLB among workers with flexible work arrangements.

## Figures and Tables

**Table 1:** All independent variables, showing variable name, scale (range and direction of the scale), references to prior studies using the variable (if applicable), construction of the variable (i.e., index or single question), and Cronbach alpha (CA) of the indices.

Variables	Scale	Reference	Construction	CA
**Organizational factors**				
Work arrangement	Flextime/non-regulated working hours	Customized	One question	
Information about organizing work	0 to 4; increasing information	Customized	Index: four questions	0.83
Unclear guidelines for flexible work	0 to 4; increased uncertainty	Customized	Index: two questions	0.81
Office type	Cell-office/activity-based office/open plan office	Customized	One question	
**Leadership behaviour**				
Relation-oriented leadership	1 to 6; increased relation-oriented leadership	[31,50,51]	Index: five questions	0.88
Structure-oriented leadership	1 to 6; increased structure-oriented leadership	[31,50,51]	Index: five questions	0.85
Change-oriented leadership	1 to 6; increased change-oriented leadership	[31,50,51]	Index: five questions	0.90
**Psychosocial factors**				
Expectations to work more than agreed	0 to 4; increased expectations	Customized	Index: four questions	0.80
Expectations of availability	0 to 4; increased expectations	Customized	Index: seven questions	0.86
Clarity of expectations about availability	0 to 4; increased clarity of expectations	Customized	Index: three questions	0.86
Quantitative job demands	1 to 5; increased job demands	[43]	Index: four questions	0.82
Influence at work	1 to 5; increased influence	[43]	Index: four questions	0.71
Social community at work	1 to 5; increased social community	[43]	Index: three questions	0.81
Social support from colleagues	1 to 5; increased social support	[43]	Index: three questions	0.72
Culture encouraging flexible work	0 to 4; increased encouraging culture	Customized	One question	
**Individual behaviour**				
Overtime work (hours/week)	Continuous; difference between the actual working hours and a normal working week	Customized	One question	
Over-commitment	1 to 4; increased over-commitment	[44]	Index: six questions	0.83
Boundary management	0 to 4; increased boundary management	[52]	Index: three questions	0.87
Interaction variable				
Perceived flexibility	0 to 4; increasing flexibility	[10]	Index: four questions	0.80

**Table 2:** Mean and SD between participants of work-life balance (WLB), perceived flexibility, occupational factors, and individual behaviours, (n, number of participants to the question or index, cf. Table 1; %, n in percent of participants).

Variables	Scale	n	%	Mean (SD)
**Organizational factors**				
Non-regulated working hours	Categorical	2073	71.2	/
Flextime	Categorical	838	28.8	/
Information about organizing work	0 to 4	2948	/	2.03 (0.97)
Unclear guidelines for flexible work	0 to 4	2938	/	1.74 (1.17)
Cell-office	Categorical	1112	38.7	/
Activity-based office	Categorical	792	27.5	/
Open plan office	Categorical	973	33.8	/
**Leadership behaviour**				
Relation-oriented leadership	1 to 6	2937	/	4.79 (0.98)
Structure-oriented leadership	1 to 6	2930	/	4.01 (0.96)
Change-oriented leadership	1 to 6	2924	/	4.15 (1.12)
**Psychosocial factors**				
Expectations to work more than agreed	0 to 4	2958	/	1.10 ( 0.92)
Expectations of availability	0 to 4	2958	/	1.07 (0.78)
Clarity of expectations about availability	0 to 4	2888	/	1.95 (1.10)
Quantitative job demands	1 to 5	2955	/	2.92 (0.72)
Influence at work	1 to 5	2956	/	3.01 (0.66)
Social community at work	1 to 5	2951	/	4.23 (0.65)
Social support from colleagues	1 to 5	2951	/	3.43 (0.70)
Culture encouraging flexible work	0 to 4	2921	/	2.13 (1.15)
**Individual behaviour**				
Overtime work (hours/week)	Continuous	2 685	/	2.74 (4.11)
Over-commitment	1 to 4	2 953	/	2.31 (0.66)
Boundary management	0 to 4	2 948	/	3.02 (0.88)
**Interaction variable**				
Perceived flexibility	0 to 4	2 952	/	2.67 (0.90)
**Dependent variable**				
Work-life balance	0 to 4	2 960	/	2.93 (1.00)

**Table 3:** Associations between occupational factors and individual behaviours, and work-life balance (WLB). Results in terms of B, standard error of B, and contribution to explained variance (∆R^2^) of multiple linear regression analyses addressing main effects (model 2) and interactions with perceived flexibility (model 3). All models were adjusted for age, gender, level of education, years of employment, marital status, children at home, and position (manager or employee).

Variables	Scale	n	Model 2. Main Effects				Model 3. Interaction Effects			
			B	SE	P	∆R^2^	B	SE	P	∆R^2^
**Organizational factors**										
Work arrangement Flextime (reference: non-regulated working hours)	Categorical	2911	0.08	0.05	0.077	0.001	0.22	0.05	<0.001	0.008
Information about organizing work	0 to 4	2948	0.31	0.02	<0.001	0.089	−0.06	0.02	<0.001	0.003
Unclear guidelines for flexible work	0 to 4	2938	−0.19	0.02	<0.001	0.050	0.01	0.02	0.583	<0.001
Office type (reference: cell-office)	Categorical	2877								
Activity-based office	Categorical		−0.03	0.05	0.453	<0.001	0.08	0.05	0.118	0.001
Open plan office	Categorical		0.02	0.05	0.645	<0.001	−0.01	0.05	0.912	0.001
**Leadership behaviour**										
Relation-oriented	1 to 6	2937	0.28	0.02	<0.001	0.071	−0.02	0.02	0.226	<0.001
Structure-oriented	1 to 6	2930	0.25	0.02	<0.001	0.054	−0.08	0.02	<0.001	0.006
Change-oriented	1 to 6	2924	0.19	0.02	<0.001	0.044	−0.03	0.02	0.022	0.001
**Psychosocial factors**										
Expectations to work more than agreed	0 to 4	2958	−0.34	0.02	<0.001	0.088	0.07	0.02	<0.001	0.004
Expectations of availability	0 to 4	2958	−0.44	0.02	<0.001	0.105	0.10	0.02	<0.001	0.005
Clarity of expectations about availability	0 to 4	2888	0.08	0.02	<0.001	0.008	0.02	0.02	0.203	<0.001
Quantitative job demands	1 to 5	2955	−0.67	0.02	<0.001	0.227	0.02	0.02	0.285	<0.001
Influence at work	1 to 5	2956	0.38	0.03	<0.001	0.059	0.02	0.03	0.694	<0.001
Social community at work	1 to 5	2951	0.41	0.03	<0.001	0.070	0.00	0.03	0.913	<0.001
Social support from colleagues	1 to 5	2951	0.41	0.03	<0.001	0.080	−0.08	0.03	0.002	0.003
Culture encouraging flexible work	0 to 4	2921	0.11	0.02	<0.001	0.015	0.02	0.02	0.303	<0.001
**Individual behaviour**										
Overtime work (hours/week)	Continuous	2685	−0.08	0.01	<0.001	0.091	0.02	0.01	0.001	0.003
Over-commitment	1 to 4	2953	−0.87	0.02	<0.001	0.317	0.02	0.03	0.552	<0.001
Boundary management	0 to 4	2950	0.63	0.02	<0.001	0.316	0.01	0.02	0.802	<0.001
Interaction variable										
Perceived flexibility	0 to 4	2952	0.29	0.02	<0.001	0.065				

Note. Three hierarchical models were constructed for each predictor: model 1 included all covariates; model 2 addressed the main effect of the occupational factor/individual behaviour shown in the left-most column; and model 3 even included the interaction effect of that factor and perceived flexibility. Abbreviations: ∆R^2^, change in explained variance relative the preceding model; B, beta coefficient; SE, standard error of B.

## Supplementary Materials

The following are available online at https://www.mdpi.com/1660-4601/17/4/1418/s1, Table S1: Description of included questions in all independent variables used in the present study.
