# Supplementary material for: Occupational and Individual Determinants of Work-life Balance among Office Workers with Flexible Work Arrangements
Source: Int J Environ Res Public Health. 2020 Feb 22;17(4):1418. doi: 10.3390/ijerph17041418 (PMC7068342; doi:10.3390/ijerph17041418)
Supplement: Supplementary file 1 [file ijerph-17-01418-s001.pdf]

Supplementary material

Occupational and Individual Determinants of Work-life Balance among Office Workers with Flexible Work Arrangements

Table 1. Description over included questions in all independent variables, used in the present study.

Variables	Scale	Included questions
Organizational factors		
Work arrangement	Categorical; Flex-time/ non-regulated working hours / other work solution	Which employment status do you have?
Information about organizing work	0 (not at all) to 4 (to a very high extent)	Do you feel that the Swedish Transport administration (STA) has given you information, (e.g. through your manager), about:<br>1) How to handle advantages and disadvantages with flexible work?<br>2) What tasks to prioritize when your time is insufficient?<br>3) What result you should achieve with your work?<br>4) Special ways on how your work should be performed and if so, how?
Unclear guidelines for flexible work	0 (completely incorrect) to 4 (completely correct)	In my work-place we have:<br>1) A need for clearer guidelines from STA on work outside regular working hours (e.g. during evenings).<br>2) A need for clearer guidelines from STA on work at other places than the office (e.g. from home).
Office type	Categorical; Cell-office /activity based office /open plan office	What type of office do you work in?
Leadership behavior		
Relation-oriented leadership	1 (completely incorrect) to 6 (completely correct)	1) My manager is considerate.<br>2) My manager shows regard for us employees as individuals.<br>3) My manager delegates responsibility and authority.<br>4) My manager socializes with us employees to build relationships.<br>5) My manager trust us employees.
Structured-oriented leadership	1 (completely incorrect) to 6 (completely correct)	1) My manager formulates clear and substantial goals for the work.<br>2) My manager allocates work to groups and individuals in a clear way.<br>3) My manager closely follows-up and controls performed work.<br>4) My manager gives clear instructions on how to do the work.<br>5) My manager makes a point of following rules and principles.

Change-oriented leadership	1 (completely incorrect) to 6 (completely correct)	1) My manager tests new work methods to improve the ability to achieve our organizational goals.<br>2) My manager has and communicates visions of the future.<br>3) My manager encourages development, innovation and learning.<br>4) My manager encourages discussions of new ideas and suggestions.<br>5) My manager starts development projects.
Psychosocial factors		
Expectations to work more than agreed	0 (not at all) to 4 (to a very high extent)	1) Do your colleagues expect you to work more than agreed?<br>2) Does your manager expect you to work more than agreed?<br>3) Do you expect yourself to answer e-mails, phone calls and/or text messages outside regular working hours?<br>4) Do you expect yourself to work more than agreed?
Expectations of availability	0 (not at all) to 4 (to a very high extent)	How well do the following questions fits you and your work situation?<br>1) Do you experience expectations of availability from your manager or colleagues during evenings after regular working hours?<br>2) Are there expectations from my colleagues that I should reply to e-mails, telephone calls and/or SMS outside regular working hours?<br>3) Do you experience expectations of availability from your manager or colleagues during non-working days?<br>4) Do you experience expectations of availability from your manager or colleagues in the morning before regular working hours?<br>5) Do you experience expectations of availability from your manager or colleagues during vacation or parental leave?<br>6) Do you experience expectations of availability from your manager or colleagues during breaks at work?<br>7) Are there expectations from my manager that I should answer emails, telephone calls and/or SMS outside reg. working hours?
Clarity of expectations about availability	0 (not at all) to 4 (to a very high extent)	1) Are my colleagues clear with their expectations of answers outside regular working hours?<br>2) Is my manager clear with expectations of answers outside regular working hours?<br>3) Am I clear with my expectations of answers outside regular working hours?
Quantitative job demands	1 (never/almost never) to 5 (always)	1) Do you get behind with your work?<br>2) How often do you not have time to complete all your work tasks?<br>3) Do you have enough time for your work tasks?<br>4) Is your workload unevenly distributed so it piles up?
Influence at work	1 (never/almost never) to 5 (always)	1) Can you influence the amount of work assigned to you?<br>2) Do you have any influence on what you do at work?<br>3) Do you have a large degree of influence concerning your work?<br>4) Do you have a say in choosing who you work with?
Social community at work	1 (never/almost never) to 5 (always)	1) Do you feel part of a community at your place of work?<br>2) Is there a good atmosphere between you and your colleagues?<br>3) Is there good co-operation between the colleagues at work?
Social support from colleagues	1 (never/almost never) to 5 (always)	1) How often do your colleagues talk with you about how well you carry out your work?<br>2) How often are your colleagues willing to listen to your problems at work?<br>3) How often do you get help and support from your colleagues?

Culture encouraging flexible work	0 (completely incorrect) to 4 (completely correct)	In my workplace, we have a culture encouraging flexible work.
Individual behavior		
Overtime work (hours/week)	Assessed in numbers rounded to the closest integer (calculated as the difference between the actual working hours and a normal working week (usually 40 working hours according to contracts)	Estimate how many hours you work per week.
Over commitment	1 (completely incorrect) to 4 (completely correct)	1) I get easily overwhelmed by time pressure at work.<br>2) As soon as I get up in the morning I start thinking about work problems.<br>3) When I get home, I can easily relax and “switch off” work.<br>4) People close to me say I sacrifice too much for my job.<br>5) Work rarely lets me go, it is still on my mind when I go to bed.<br>6) If I postpone something that I was supposed to do today I’ll have trouble sleeping at night.
Boundary management	0 (completely incorrect) to 4 (completely correct)	I can by myself:<br>1) Control if I can keep my work and my privacy separate<br>2) Control if I have clear boundaries between my work and my private life<br>3) Control if I can combine my work and my privacy during the day and week
Interaction variable		
Perceived flexibility	0 (completely incorrect) to 4 (completely correct)	I can by myself:<br>1) Decide where I perform my work<br>2) Decide what time I work a certain day<br>3) Decide when different tasks should be performed<br>4) Decide how my work should be performed
